# Crying Therapy Intervention for Breast Cancer Survivors: Development and Effects

**DOI:** 10.3390/ijerph17134911

**Published:** 2020-07-07

**Authors:** Hye-Sun Byun, Hyenam Hwang, Gyung-Duck Kim

**Affiliations:** 1 School of Nursing, Yeungnam University College, Daegu 42415, Korea; bbhhsun@ync.ac.kr; 2 Department of Nursing, Daegu University, Daegu 42400, Korea; 3 Department of Nursing, Dongyang University, Kyungpook 36040, Korea; gdkim@dyu.ac.kr

**Keywords:** crying therapy intervention, breast cancer, quasi-experiment, stress alleviation, positive emotional change

## Abstract

Background: crying therapy is currently being applied in some countries to treat cancer patients, manage pain, and promote mental health. However, little nursing and medical research on the effects of crying therapy has been conducted in other parts of the world. This study aimed to develop a crying therapy program for breast cancer survivors and assess its effects. Interventions/method: data from 27 breast cancer survivors in South Korea were analyzed. The intervention, employing a single group, pre-post-test quasi-experimental design, was divided into three phases, and effects were verified for emotional (distress, fatigue, and mood conditions) and physiological (cortisol, immunoglobulin G, and blood pressure) variables. Results: there were significant changes in distress, mood changes, and immunoglobulin G and smaller changes in blood pressure postintervention. Fatigue and cortisol showed no significant changes. Conclusions: this study demonstrated the effectiveness of a short-term crying therapy program that can induce positive emotional changes and physiological effects in breast cancer survivors. This intervention can improve quality of life, indicating its value as a self-care program for cancer survivors.

## 1. Introduction

Breast cancer is the leading cancer among females, with a global incidence of 24.2% [1]. It is also the cancer with the highest survival rate (90.0%) [1]. Various medical procedures such as surgery, chemotherapy, antihormone therapy, and radiotherapy are contributing to increasing numbers of survivors. However, despite their therapeutic effects, these treatments induce stress, causing patients to experience various stress response symptoms such as increased blood pressure (BP), anxiety, low mood, and immunosuppression [2,3]. As these responses can be detrimental to the normal course of treatment, it is important to identify effective measures to alleviate stress.

In addition to hampering treatment, stress experienced by most cancer patients can exacerbate symptoms, and unmanaged distress can have negative effects on their quality of life (QOL) [4]. The adverse effects of breast cancer treatment also include mood disorder symptoms such as anxiety, depression, anger, fatigue, and loss of energy. Often, these symptoms persist even after the conclusion of treatment and can continue to impair QOL [5,6]. Therefore, to improve QOL in patients with breast cancer, it is important that interventions target not only physical health but also reduce negative psychological symptoms [7].

As a physiological symptom experienced by breast cancer survivors, stress has negative effects on health via neural and endocrinological pathways. The stress hormone, cortisol, is secreted by the adrenal cortex and affects immune responses by suppressing the metabolism and cytotoxicity through T lymphocytes and natural killer cells [8]; thus, high stress levels are associated with increased cortisol levels [9]. When humans experience chronic stress, the immune system is inhibited, and immunoglobulin production declines [10]. Specifically, immunoglobulin G (IgG) is responsible for immunity against various pathogens, including bacteria, viruses, and toxins, and plays an important role in secondary immune responses [11]. IgG is an important biomarker for predicting disease course and monitoring responses to treatment in patients with breast cancer experiencing chronic stress [11]. When breast cancer survivors are exposed to long-term stress, immunoglobulin production decreases, limiting antibody responses, which can negatively affect survival and recovery rates [11,12]. It is, therefore, necessary to implement interventions to relieve stress and reduce stress hormones, thereby increasing immunoglobulin production.

Crying therapy has recently been reported to be effective for relieving stress [13,14,15,16] and improving immune system functioning [12,17]. It uses weeping as a supplement to other therapeutic interventions to reduce physical and emotional stress [12,18]. Specifically, crying facilitates self-reflection, forgiveness toward the self and others, freedom from things holding one back, peace of mind, and gratitude for small things in life [12,15]. Previous studies have shown that the positive effects of crying include stimulating the sympathetic nervous system to increase blood circulation, respiratory rate, and oxygen utilization, increasing immune system activity, reducing stress and pain through increased secretion of neurotransmitters such as endorphins, enkephalins, and serotonin, and stimulating natural killer cells to fight cancer [12,14,17,19].

Additionally, crying promotes catharsis and emotional healing when used by the patient and family members for managing stress, improves mood by stimulating endorphin secretion, alleviates pain, and has positive effects on emotional well-being [20,21,22,23,24,25]. In Lee’s study [15], crying was used with cancer patients in a clinical setting for the first time in South Korea, and the findings demonstrated an increased survival rate, a cathartic effect, and the effectiveness of crying as a natural therapeutic agent. Similarly, previous studies have reported that crying helps individuals adapt physically and emotionally to stressful situations [12,26], reinforces therapeutic relationships, and enhances the treatment process [21,26]. Currently, in the Netherlands, crying therapy is being used by psychologists for patients with terminal cancer, diseases causing severe pain, and mental health issues [21,27]. However, experimental studies on the effects of crying therapy in nursing and medical research worldwide are scarce. Thus, this study aimed to develop a crying therapy program for breast cancer survivors as well as to investigate its effectiveness.

### Objective and Hypotheses

The objective of this study was to develop a crying therapy program for breast cancer survivors and to investigate its emotional and physiological effects.

**Hypothesis 1** **(H1).** *Participants in the Crying Therapy Program Will Show Emotional Effects*.

Hypothesis 1-1. Participants will show reduced distress scores.

Hypothesis 1-2. Participants will show reduced fatigue scores.

Hypothesis 1-3. Participants will show reduced mood scores (depression, anger, and anxiety).

**Hypothesis 2** **(H2).** *Participants in the Crying Therapy Program Will Show Physiological Effects*.

Hypothesis 2-1. Participants will show reduced blood cortisol levels.

Hypothesis 2-2. Participants will show increased blood IgG levels.

Hypothesis 2-3. Participants will show changes in BP.

## 2. Materials and Methods

### 2.1. Design

**Figure 1:**
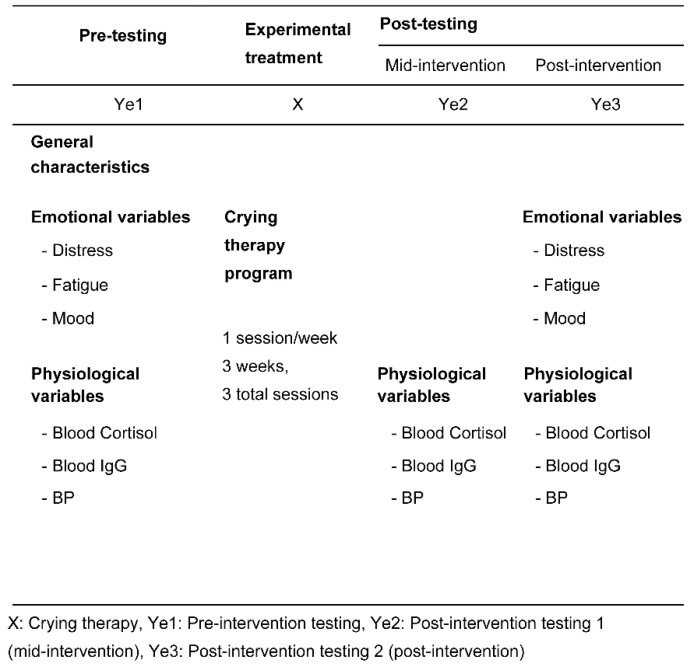
Research design.

A crying therapy program was developed, and a one-group time-series design was used to test its emotional and physiological effects in breast cancer survivors (Figure 1).

### 2.2. Sample and Setting

**Figure 2:**
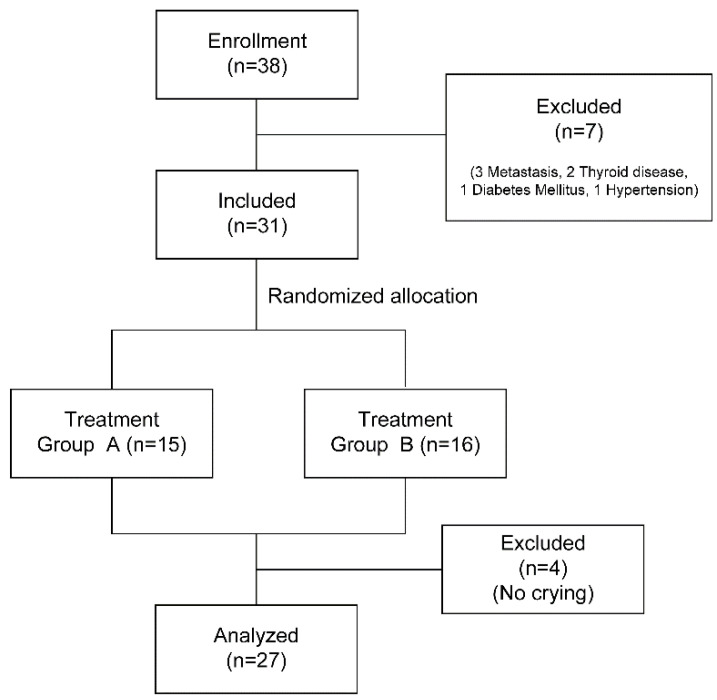
Flow diagram of sampling.

Participants were a random sample of breast cancer survivors who were active in a self-help group in D City, South Korea. Using G*Power 3.1.7 (Heinrich-Heine-Universität Düsseldorf, Düsseldorf, Germany), for an effect size of 0.25, a significance level of 0.05, and power of 0.80, it was determined that a sample size of 24 participants was required. Although a total of 38 applicants were recruited, the intervention was conducted with 31 participants after excluding seven applicants who did not fulfill the selection criteria. Data from 27 participants were used for the final analysis after excluding four participants who did not cry during the intervention (Figure 2).

The participant inclusion criteria included females aged 18 years and older who did not show metastasis of breast cancer; did not have endocrinological disorders (e.g., diabetes), hypertension, or immune disorders; and who had not used mood-affecting medication (e.g., antidepressants, anxiolytics, etc.) within the four weeks preceding recruitment. 

#### 2.2.1. Variables

##### Emotional Effects

Distress. The U.S. National Comprehensive Cancer Network’s [28] Distress Thermometer (DT) and Problem List (PL) for Patients, which have demonstrated reliability and validity [29], were used to measure distress. The DT is a visual analog scale in the form of a thermometer ranging from 0 to 10 points, where a higher score corresponds to a higher level of distress. The PL comprises a total of 39 “Yes” or “No” questions across five domains: practical problems, family problems, emotional problems, spiritual/religious concerns, and physical problems. The instrument has an internal consistency reliability of 0.86 [30], and, in this study, the reliability coefficients were 0.88 and 0.89 in the preintervention and postintervention tests, respectively.

Fatigue. Fatigue was measured using the Korean version of the Functional Assessment of Chronic Illness Therapy-Fatigue Scale developed by FACIT [31]. The instrument comprises 13 questions, with higher scores indicating higher fatigue levels. In a prior study with South Korean patients with breast cancer, this instrument showed a Cronbach’s alpha of 0.91 [32], and, in this study, the reliability coefficients were 0.87 and 0.86 in the preintervention and postintervention tests, respectively.

Mood. As an assessment of mood, levels of depression, anger, and anxiety were measured using the Emotion Thermometers tool developed by Mitchell [29]. Participants rated their current levels of depression, anger, and anxiety on a scale ranging from 0 to 10 points, with higher scores indicating higher levels of depression, anger, and anxiety.

##### Physiological Effects

Serum cortisol. To measure cortisol levels, 3cc of venous blood was collected and tested using a radioimmunoassay (RIA: Roche E-170).

Immune system activity (serum IgG). RIA (Roche COBAS Integra 800) was used to measure serum IgG.

BP. BP was measured using an aneroid sphygmomanometer.

### 2.3. Experimental Intervention Program: Crying Therapy

The three-phase crying therapy program was developed through a literature review [15,33,34], discussions among the researchers, and an expert panel review. The program comprised an introductory, execution, and closing phase, each of which lasted two hours. The program’s contents and implementation were reviewed by two experts in crying therapy for patients with cancer (Table 1).

#### 2.3.1. Phase 1: Introductory Phase

Phase 1 was a preparatory phase consisting of an introduction to the program, the researchers, and psychoeducation on the effects of crying therapy.

#### 2.3.2. Phase 2: Execution Phase

Phase 2 was divided into four stages: (1) finding my wounded self, (2) finding another me, (3) treating my wounded self, and (4) self-discovery of hope. The “finding my wounded self” stage focused on alleviating physical and mental tension through abdominal breathing, progressive muscle relaxation, and imagery training. The “finding another me” stage included silent contemplation of distress to promote emotional involvement and of photographs of the participants at different ages, which they were asked to prepare ahead of time. The “treating my wounded self” stage was the main crying stage, and comprised watching videos, silently contemplating cleansed wounds, writing a will, and reading the will aloud. This stage provided sufficient time to help the participants cry comfortably. Finally, the “self-discovery of hope” stage comprised silent contemplation of the positive aspects about themselves that they discovered during the first three stages of the execution phase.

#### 2.3.3. Phase 3: Closing Phase

Phase 3 consisted of silent contemplation, writing a letter to oneself, and an opportunity to share the emotions one had experienced during Phases 1 and 2. After this phase of the program, the letters that participants wrote to themselves were sent to them via mail.

### 2.4. Study Procedures

#### 2.4.1. Ethical Considerations

The study was approved by the D. University Institutional Review Board (Approval number: 1041495-201702-HR-01-01) and conformed to the standards of the Declaration of Helsinki. Prior to the intervention, the study’s objectives and methods were explained to the administrators of a self-help group in D City. After receiving their approval, advertisements were posted at the self-help group center to recruit participants. The study’s objective and crying therapy intervention were explained to the participants, and they were informed about the need to collect blood samples on three occasions before being asked to sign the participation consent form. It was further explained to the participants that their privacy and anonymity would be protected and that they could refuse to participate or withdraw from the study at any time. Participants were given a small payment as compensation for participation.

#### 2.4.2. Preliminary Study

For the preliminary study, recruitment advertisements were posted at the self-help group center for one week starting on 24 April 2017. We received applications from three breast cancer survivors who met the inclusion criteria of the main study, and the crying therapy program was administered to them for three weeks from 9 May to 23 May 2017. During this process, the total duration of the program and any discomfort or difficulties experienced by the participants were noted. Additionally, pre- and postintervention tests were administered for the dependent variables. Once it was confirmed that there were no issues with the program, the main intervention commenced.

#### 2.4.3. Main Study

The main study was conducted every week and lasted three weeks from 3–20 July 2017. To recruit participants, advertisements were posted for 30 days before commencing the study. The intervention program was conducted in a seminar room at the university of one of the researchers. The 31 participants were randomly divided into two groups of 15 and 16 participants each to create an effective environment for the experiment. The experimental treatment of each group was conducted using the same mediator in the same way. To measure the physiological variables, blood samples of program participants in each phase were collected by the three researchers and two clinical pathologists at approximately the same time of the day to minimize fluctuations caused by circadian rhythms.

Questionnaires to examine participants’ emotional states were administered before Phase 1 and after Phase 3. To examine physiological variables, blood was collected before Phase 1 and after Phases 2 and 3. After each phase, the participants were provided with snacks and tea.

### 2.5. Data Analysis

The collected data were analyzed using PASW 24.0 (IBM Crop, Armonk, NY, USA). General and disease-related characteristics were analyzed using descriptive statistics. Paired *t*-tests were performed to test the hypotheses examining the program’s effects on emotional variables. Repeated-measures ANOVAs and Bonferroni analyses were used to test the hypotheses regarding the program’s effects on physiological variables.

## 3. Results

### 3.1. Participant Characteristics

The participants’ mean age was 58.4 years (SD = 6.45, range: 47–70), 59.3% had graduated from high school, and 85.2% were married. Regarding disease-related characteristics, 48.1% of the participants had stage 2 cancer at the time of diagnosis, 51.9% of the participants had undergone a partial mastectomy, and two participants reported a family history of breast cancer (the maternal aunt, in both cases) (see Table 2).

### 3.2. Hypothesis Testing

#### 3.2.1. Hypothesis 1

Distress scores decreased significantly at postintervention testing (3.74 ± 2.54) as compared to preintervention testing (4.63 ± 1.96; *t* = 2.884, *p* = 0.008). Distress PL scores also decreased significantly postintervention (7.63 ± 6.66) as compared to preintervention (9.93 ± 6.82). These results supported Hypothesis 1-1. Fatigue scores did not differ significantly between pre- and postintervention, leading to the rejection of Hypothesis 1-2. Depression scores decreased significantly postintervention (3.04 ± 2.26) as compared to preintervention (4.15 ± 2.66; *t* = 3.162, *p* = 0.004). Similarly, anger scores decreased significantly postintervention (2.67 ± 2.11) as compared to preintervention (3.89 ± 2.59; *t* = 2.877, *p* = 0.008), and anxiety scores also decreased significantly postintervention (2.85 ± 2.91) as compared to preintervention (3.93 ± 2.75; *t* = 3.108, *p* = 0.005). These results supported Hypothesis 1-3 (Table 3).

#### 3.2.2. Hypothesis 2

Cortisol levels did not show any significant differences between pre-, mid-, and postintervention testing, leading to the rejection of Hypothesis 2-1. IgG levels (mg/dl) increased significantly over time (preintervention: 1268.37 ± 217.78, midintervention: 1307.70 ± 232.80, postintervention: 1346.96 ± 235.84; F = 24.775, *p* < 0.001), supporting Hypothesis 2-2. Systolic blood pressure (SBP, mmHg) showed changes over time (preintervention: 126.22 ± 15.26, midintervention: 133.07 ± 14.27, postintervention: 127.00 ± 15.28; *F* = 8.703, *p* = 0.001). SBP was lower at preintervention testing as compared to midintervention (*p* = 0.001) and lower at postintervention testing as compared to midintervention (*p* = 0.009), but there was no significant difference between pre- and postintervention. Diastolic blood pressure (DBP, mmHg) showed changes over time (preintervention: 80.89 ± 10.63, midintervention: 86.00 ± 9.34, postintervention: 84.70 ± 8.69; *F* = 9.239, *p* < 0.001). DBP was lower at preintervention testing as compared to midintervention (*p* = 0.002) and postintervention (*p* = 0.024), but there was no significant difference between mid- and postintervention. Thus, Hypothesis 2-3 was partially supported (Table 4).

## 4. Discussion

In this study, we developed a crying therapy program for breast cancer survivors and tested its emotional and physiological effects. The results provided evidence to support the clinical application of the program for breast cancer survivors. There were significant decreases in distress levels measured pre- and postintervention. These results support Miles’s finding [22] that the release of stress-inducing hormones through tears was effective in reducing emotional stress, Vingerhoets et al.’s report [24] that tears helped relieve distress, and Vingerhoets and Bylsma’s [26] result that crying functioned as catharsis, aiding in emotional recovery. In this study, the reduction in the participants’ distress could perhaps be attributed to the emotional tears shed while participating in the crying therapy program.

Fatigue decreased slightly after the crying therapy program but not significantly. These results can be considered consistent with the findings of a study in which participants reported experiencing “lightening” and “sleepiness” after crying [18], and another wherein crying induced effects such as the alleviation of physical tension [10]. However, quantitative research on the effects of crying on fatigue is insufficient, making it difficult to compare the results of this study with those from the existing literature. Thus, there is a need for further research in this area.

Mood states, in this case depression, anger, and anxiety levels, decreased significantly after the program. This is consistent with previous findings. For example, Jeon [10] and Han and Kim [33] reported that crying therapy was effective in alleviating repressed emotions, depression, and anger. Additionally, Lee [15] reported that crying is a mental and emotional therapeutic agent for anger toxins and that after experiencing extreme catharsis, the mind becomes peaceful and calm. Lee [16] also reported that crying in this context is an expression of internalized anger and repression. It is worthwhile to control internalized emotions because Korean culture is reluctant to show its own feelings [35]; accordingly, it is thought that effectively handled crying would help to relieve congested anger. These results, coupled with those of this study, indicate that effective crying can help relieve repressed anger. Additionally, consistent with the results of this study, Hastrup et al. [14] reported that the cleansing effects of crying reduced depression in elderly individuals. Fooladi [18] reported that, after crying, participants experienced healing effects such as “the feeling of my spirit being purified” or “the feeling of toxins being cleansed from my body” (p. 253–254). Various researchers have reported that crying helped participants experience feelings of tranquility, relief, and emotional cleansing [16]; induced positive mood states, and alleviated tension and repressed emotions [25]; and helped alleviate anger [36]. Furthermore, crying has been found to have positive effects on emotional well-being [37,38], a finding that supports our results. Although there are various hypotheses about the positive effects of crying, supporting evidence has suffered from a severe lack of experimental studies using crying therapy both in South Korea and globally, rendering it difficult to make direct comparisons with the present findings. Therefore, we believe there is a need for more experimental studies on the emotional effects of crying therapy in breast cancer survivors as well as qualitative research to study in-depth experiential data.

Regarding the physiological effects of the crying therapy, no decrease in cortisol levels was found. This finding is consistent with a study by Vingerhoets and Bylsma [37], which found no significant change in cortisol after crying, but conflicts with reports by Lee [15] and Han and Kim [33], who suggested that as tears contain high levels of stress hormones, cortisol levels decrease after crying. In this study, there was no significant reduction in cortisol levels induced by crying therapy because the participants’ preintervention cortisol levels were within the normal range. Therefore, to examine whether cortisol is excreted through tears, a crying therapy intervention should be performed with patients who have recently been diagnosed with breast cancer, as they may have higher cortisol levels owing to higher levels of stress.

The analysis of the second physiological variable, IgG, revealed that there were significant increases in immune system activity at mid- and postintervention testing compared to preintervention. This supports a report by Lee [15] that tears are a natural defense that enhances immune system activity, increasing IgG levels over two-fold, and suppressing or reducing cancer cells. Further, these results are consistent with Han and Kim’s [33] findings that, after crying freely, participants showed reduced levels of stress hormones, increased parasympathetic nervous system activity, and elevated IgG levels. Crying has also been shown to increase the levels of natural killer cells, which destroy cancer cells in the lymphatic system as part of the immune response [33]; thus, we believe that crying therapy should be actively utilized in interventions for patients with cancer.

Finally, when the physiological effects of crying therapy on BP were analyzed, both SBP and DBP increased significantly at midintervention testing compared to preintervention testing, and subsequently decreased at postintervention testing, but there was no statistically significant difference. These results support a report by Lee [15] that, when initially shedding tears, the autonomic nervous system responds to increased blood flow by raising heart rate and BP, but after crying, stress hormones and adrenaline decrease, the parasympathetic nervous system is activated, the individual becomes calm, and BP decreases, improving cardiovascular functioning. However, the fact that there was no statistically significant change in BP in this study is thought to be because participants were not hypertensive. To examine whether a crying therapy program can reduce BP, the intervention would need to be repeated with patients with hypertension.

Breast cancer survivors experience reduced QOL because of physical and psychological distress arising from fear as a result of diagnosis, treatment-related adverse effects, or relapse. The present results provide healthcare professionals with basic knowledge and nursing practices for crying therapy. Additionally, by testing the emotional and physiological effects of crying therapy for cancer survivors, this study demonstrated that implementing crying therapy self-care programs for breast cancer survivors could be an effective evidence-based nursing intervention to improve their QOL. Although the scope of this study is limited in that it only examined a few variables, it has sufficient clinical value as a preliminary study. In terms of future research, repeated experiments using diverse variables and controls to identify other biological indicators are required.

## 5. Conclusions

This study confirmed that crying therapy is effective in improving stress levels, mood, and immune system activity in female breast cancer survivors. Further, the study demonstrated its potential utility in clinical settings as a noninvasive nursing intervention for alleviating psychological distress in patients with cancer.

This study has several limitations that could not be addressed when developing and implementing the crying therapy intervention. Firstly, because this study implemented structured crying therapy for breast cancer survivors experiencing diverse physical symptoms and did not measure various other objective physiological indicators such as T lymphocytes and natural killer cells, more scientific evidence needs to be collected to determine the effectiveness of crying therapy for general cancer patients in clinical settings. Secondly, this was a single-group study, and, unfortunately, a control group could not be established. In the future, it would be necessary to perform experiments with a control group. Finally, caution must be exercised when generalizing the findings of this study, as the participants were breast cancer survivors who were active members of a particular self-help group.

## Figures and Tables

**Table 1:** Development of crying therapy program.

Week/Phase			Program Contents	Background Music	Time (mins)
**Week 1**	**Introductory phase**	Developing intimacy, opening up, preparing for crying therapy	Exchanging greetings Explaining the effects of crying therapy Introducing the need for the program Introducing the procedure of the program		120 min
**Week 2**	**Execution phase**	“Finding my wounded self”	Breathing methods to relieve physical and psychological tension - Abdominal breathing - Progressive muscle relaxation - Imagery training		15 min
		“Finding another me”	Communal silent contemplation for emotional involvement Silent contemplation of photographs at different ages Silent contemplation on infancy Silent contemplation on preschool age Silent contemplation on school age Silent contemplation on adolescence	Sad piano pieces	25 min
		“Treating my wounded self”	Watching videos of terminal patients Writing a will Silent contemplation to cleanse wounds Reading the will aloud Hugging partners	Meditation music	70 min
		“Self-discovery of hope”	Final silent contemplation Stretching and shouting	Meditation music	10 min
**Week 3**	**Closing phase**	Sharing feelings	Introducing cases of overcoming cancer Final silent contemplation Reading poetry aloud Writing a letter to oneself Expressing one’s feelings and changes	Meditation music	120 min

**Table 2:** General participant characteristics (N = 27).

Variable	Category	*n*	%	Variable	Category	*n*	%
**Sociodemographic Characteristics**							
**Educational level**	Middle school graduation or below	5	18.5	**Household size (number of persons)**	1–2	6	22.2
	High school graduation	16	59.3		3–4	14	51.9
	University graduation or above	6	22.2		5 or more	7	25.9
**Marital status**	Married	23	85.2	**Occupation**	Unemployed	25	92.6
	Widowed	4	14.8		Employed	2	7.4
**Exercise** **(times per week)**	Once or less	4	14.8	**Religion**	Yes	21	77.8
	2–3 times	14	51.9		No	6	22.2
	4 times or more	9	33.3				
**Disease-related characteristics**							
**Stage at diagnosis**	0	3	11.1	**Time since diagnosis** (**years)**	0–5	15	55.6
	1	9	33.3		6–10	8	29.6
					11–20	4	14.8
	2	13	48.1	**Family history of breast cancer**	Yes	2	7.4
	3	2	7.4		No	25	92.6
**Surgery**	Partial mastectomy	14	51.9	**Radiotherapy**	Yes	22	81.5
	Total mastectomy	13	48.1		No	5	18.5
**Chemotherapy**	Yes	20	74.1	**Antihormone therapy**	Yes	9	33.3
	No	7	25.9		No	18	66.6

**Table 3:** Comparison of emotional variables (N = 27).

Variable		Possible Range	Pre	Post	*t* (*p*)
			M ± SD	M ± SD	
**Distress**	**Thermometer**	0–10	4.63 ± 1.96	3.74 ± 2.54	2.884 (0.008)
	**Problem List**	0–39	9.93 ± 6.82	7.63 ± 6.66	2.820 (0.009)
**Fatigue**		0–52	15.15 ± 7.28	13.70 ± 8.17	1.257 (0.220)
**Mood conditions**	**Depression**	0–10	4.15 ± 2.66	3.04 ± 2.26	3.162 (0.004)
	**Anger**	0–10	3.89 ± 2.59	2.67 ± 2.11	2.877 (0.008)
	**Anxiety**	0–10	3.93 ± 2.75	2.85 ± 2.91	3.108 (0.005)

**Table 4:** Comparison of physiological variables (N = 27).

Variable		Pre ^a^	Mid ^b^	Post ^c^	F (*p*) Bonferroni
		M ± SD	M ± SD	M ± SD	
**Cortisol (μg/dL)**		6.97 ± 2.54	7.11 ± 3.04	7.33 ± 3.90	0.176 (0.839)
**IgG** **(mg/dL)**		1268.37 ± 217.78	1307.70 ± 232.80	1346.96 ± 235.84	24.775 (<0.001) a < b (0.001); b < c (0.001); a < c (<0.001)
**Blood pressure** **(mm/Hg)**	**SBP**	126.22 ± 15.26	133.07 ± 14.27	127.00 ± 15.28	8.703 (0.001) a < b (0.001); b > c (0.009)
	**DBP**	80.89 ± 10.63	86.00 ± 9.34	84.70 ± 8.69	9.239 (<0.001) a < b (0.002); a < c (0.024)

Note: IgG: immunoglobulin G, SBP: systolic blood pressure, DBP: diastolic blood pressure.
